# Torque Teno Virus: A Promising Biomarker in Kidney Transplant Recipients

**DOI:** 10.3390/ijms25147744

**Published:** 2024-07-15

**Authors:** Sara Dal Lago, Paola Brani, Giuseppe Ietto, Daniela Dalla Gasperina, Francesco Gianfagna, Cristina Giaroni, Annalisa Bosi, Francesca Drago Ferrante, Angelo Genoni, Hafza Zahira Manzoor, Andrea Ambrosini, Marco De Cicco, Corradina Dina Quartarone, Sara Khemara, Giulio Carcano, Fabrizio Maggi, Andreina Baj

**Affiliations:** 1 Nephrology Department, ASST Sette Laghi, University of Insubria, 21100 Varese, Italy; 2 Department of Medicine and Technological Innovation, University of Insubria, 21100 Varese, Italy; 3 Department of Medicine and Surgery, University of Insubria, 21100 Varese, Italy; 4 Mediterranea Cardiocentro, 80122 Napoli, Italy; 5 Laboratory of Microbiology, ASST Sette Laghi, 21100 Varese, Italy; 6 Laboratory of Virology, National Institute for Infectious Diseases L. Spallanzani—IRCCS, 00149 Rome, Italy

**Keywords:** Torque Teno Virus, anellovirus, immunosuppression, kidney transplantation, immunosuppressive therapy, infection

## Abstract

Torque Teno Virus (TTV) is a ubiquitous component of the human virome, not associated with any disease. As its load increases when the immune system is compromised, such as in kidney transplant (KT) recipients, TTV load monitoring has been proposed as a method to assess immunosuppression. In this prospective study, TTV load was measured in plasma and urine samples from 42 KT recipients, immediately before KT and in the first 150 days after it. Data obtained suggest that TTV could be a relevant marker for evaluating immune status and could be used as a guide to predict the onset of infectious complications in the follow-up of KT recipients. Since we observed no differences considering distance from transplantation, while we found a changing trend in days before viral infections, we suggest to consider changes over time in the same subjects, irrespective of time distance from transplantation.

## 1. Introduction

Several viral agents other than the major known pathogenic viruses are found in clinical specimens. Referred to as the “human virome”, they are essential components in maintaining human health. Torque Teno Virus (TTV) is a circular, single-stranded, non-enveloped, negatively polarized DNA virus measuring between 3.4 and 3.9 Kb in length and 30–32 nm in diameter. As a member of the family *Anelloviridae*, which is divided into 30 genera and 155 species, TTV belongs to the genus *Alphatorquevirus* [1]. To date, seven genotypes have been isolated. Genotypes 1, 2, 3, 4, and 5 appear to be widespread worldwide, while group 7 has been found at least in both Taiwan and China, and group 6 only in Taiwan [2].

Discovered in 1997, TTV is now considered ubiquitous, with a prevalence that varies by geographical area and can exceed 90% of the adult population [3,4,5]. No known disease has been associated with TTV [6], and it has been hypothesized that the entire human population is infected with TTV, often with multiple species simultaneously. The first infection occurs in young children; it is still unclear whether this is symptomatic or not. Thereafter, the virus probably remains latent, although no site of latency has been identified. Other hypotheses are that the virus remains in a persistent chronic form or that continuous reinfections occur when immune defenses are lowered [6], but not much is known about the interaction between TTV and the immune system [7,8].

After the hypothesis of a possible pathogenic role for TTV was rejected, TTV attracted attention as a possible marker of immunosuppression [9] since the viral load of TTV was inversely correlated with the competence of the immune system [10]. In fact, hosts with compromised immune function due to a variety of aetiologies, including sepsis [11], HIV infection [12,13,14,15], cancer [16,17], bone marrow [18,19,20], and solid organ transplantation (SOT) have, on average, higher TTV viral loads than the general population.

Kidney transplantation (KT) is the gold standard for the treatment of end-stage renal disease (ESRD). The immunosuppressive drug regimen plays a key role in KT, reducing rejection rates and preventing the formation of donor-specific antibodies (DSA). However, a high immunosuppressive burden increases the patient’s susceptibility to both opportunistic infections [21] and cancer [22]. As immune status plays a key role in determining KT outcomes, estimating the optimal immunosuppressive load is one of the major challenges in the clinical management of SOT recipients [23]. The current approach is based, for some immunosuppressants, on monitoring the plasma drug load over time. Although this approach has contributed to increased graft and patient survival over the past 20 years, it has inherent limitations as there is no universal optimal dose because several factors play a role in determining the therapeutic window, including the association with other immunosuppressive agents, time since KT, and immunologic risk. In addition, maintaining the therapeutic window is complicated by the large interindividual pharmacokinetic differences in the metabolism of immunosuppressive drugs [24] and it does not appear to play a role in the long-term prediction and prevention of chronic rejection and adverse events [25].

The infection rate after SOT varies over time; it is highest in the first three months after transplantation and then gradually decreases, and immunosuppressive therapy is the main risk factor for infectious complications [21,26,27]. Among the most important pathogenic viruses in the immunocompromised host are BK polyomavirus and cytomegalovirus (CMV) [28,29]. Bacterial infections also affect KT recipients more frequently than the general population: UTIs are particularly common, with the most relevant pathogens being *Enterococcus faecalis*, *Escherichia coli*, *Pseudomonas aeruginosa*, and *Klebsiella pneumoniae*. Infections in these patients are difficult to detect and treat for several reasons, including the fact that symptoms are often masked by the administration of immunosuppressive therapy, that a greater number of different pathogens may be responsible for the infection (including opportunistic pathogens), and that there may be interactions between antimicrobial and immunosuppressive therapy. Early detection is of paramount importance to promptly initiate treatment and prevent further complications, including graft loss and death [21,30].

In conclusion, there is a need in the management of SOT to identify a marker that overcomes the inherent limitations of the currently used methods for assessing the level of immunosuppression and predicting infection, and that could be used independently or in combination with them [31]. Ideally, this marker should be able to detect both excessive and inadequate immunosuppression, be standardized, and be easy to implement in routine follow-ups [32]. Currently, several markers have been proposed, mostly based on specific elements that play different roles in the immune response, but none of them have been validated and actively used in clinical practice. These markers provide a limited picture of immune status due to test specificity; the direction that research is attempting to take is to instead use a marker that would reflect overall immune function. Although TTV plasma levels are mainly controlled by T cells, the control is also partly mediated by other components of the immune response, reflecting T lymphocyte function and overall host immune function [33].

Immediately following SOT, an increase in total human viral load and genotypic diversity is observed [30,34]. Specifically, anelloviruses become predominant and make up 94% of the human virome [30] and TTV seropositivity rises to 100% [35]. Assessment of TTV viremia appears to better characterize a patient’s immunosuppression status than current methods, as viral load appears to be associated with the dose and type of immunosuppressive drug [9,36,37], but also with both short- and long-term adverse effects, such as acute graft rejection, higher infection rate, chronic graft rejection, and death [38,39,40,41,42]. The precise range of TTV concentrations at which optimal immune function is achieved remains unclear. While several studies have identified different cut-offs at different time points, there is no conventionally accepted cutoff [43]. Although the role of plasma (pTTV) in SOT recipients has been investigated in several studies, there are currently no data on the potential role of monitoring urinary TTV load (uTTV) in these immunosuppressed patients.

TTV levels also appear to vary according to pre-transplant characteristics of KT recipients (such as age at transplantation, gender, pre-KT dialysis [44,45,46,47], and underlying disease [48]), although their role is controversial. Most of the available studies show an association between age and TTV viral load [49,50], which can be explained by the fact that in the elderly there is a reduction in the efficiency of the immune system, known as immunosenescence [51,52].

The aim of our study is to validate TTV as a marker of immune status in KT recipients, to evaluate TTV plasma and urine kinetics from pre-transplant to 150 days after KT, to investigate the relationship between inter-individual differences in viral load and clinical factors or recipient characteristics at baseline, and to evaluate the associations between TTV and infectious events.

## 2. Results

### 2.1. Baseline Characteristics

The characteristics of the 42 enrolled patients are shown in Table 1 and Table 2. The mean ± SD of the actual duration of follow-up was 103.4 ± 38.4 days.

The cohort of patients recruited had similar characteristics (e.g., age at transplantation, sex, and time on dialysis) to the population of KT recipients from the National Transplant Center. The most common underlying diseases we found in the recruited patients were glomerular diseases (28.6%) and cystic diseases (19%), followed by diabetic nephropathy, hypertensive nephrosclerosis, nephrectomy, chronic pyelonephritis in vesicoureteral reflux (VUR), and other syndromes. Most patients had a positive CMV serostatus at baseline (88%), received the organ from a cadaver donor (81%), and had never received a kidney transplant before (83%). More than half of the patients (69%) received basiliximab in the induction phase, while the remaining patients (31%) received double induction with basiliximab and thymoglobulin; Table 3 provides a detailed overview of the immunological characteristics of the patients. Finally, 14 subjects (33.3%) experienced a delayed graft function after transplant, defined as the need for substitutive therapy (dialysis) in 7 days after KT. These events demonstrated no correlation with pTTV and uTTV.

In 26 of 42 patients, a blood sample was obtained prior to KT to quantify pre-KT viremia (pTTV at T_0_). The presence of TTV DNA was detected in each sample collected, with a mean viral load of 5.50 copies/mL. In 7 of the 42 patients, a urine sample was also collected prior to KT: TTV DNA copies were detected in three samples.

Correlations between variables are reported in Supplementary Table S1. Regression analyses (age and sex as covariates) showed that pTTV levels at T_0_ were associated with dialysis years (for each increase in dialysis year, DNA copies increased by 0.21 ± 0.07 SE, *p* = 0.005) (Table 4).

### 2.2. TTV Kinetics after KT

**Figure 1:**
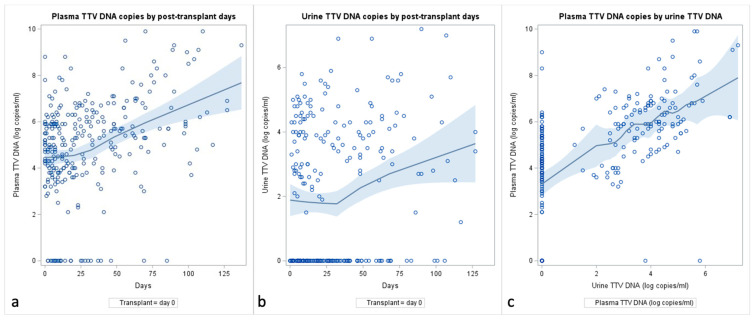
Changes in mean levels of plasma (panel **a**) and urine (panel **b**) TTV during follow-up. (Panel **c**): Change in pTTV by uTTV. Locally weighted regression (PROC SGPLOT with LOESS statement in SAS) using a scatterplot smoothing method that automatically determines the optimal smoothing parameter.

We then analyzed the associations between TTV levels over time post-transplantation and their potential determinants. TTV DNA levels were measured at baseline and during follow-up (n = 363 measurements). Immediately after KT, the pTTV load (medium at day 15: 4.54 ± 1.77 log copies/mL) was lower than immediately before KT (medium: 5.26 ± 1.31 log copies/mL), with a later increase after month 1 (Table 5). An association between time elapsed since KT and TTV emerged is as follows: both pTTV and uTTV increased 0.02 log copies/mL per day post-KT (SE = ±0.004, *p* < 0.0001; Table 6). The increase was evident after the first month (Figure 1a,b): +0.021 log copies/mL (SE = 0.005, *p* < 0.0001) and +0.029 log copies/mL (SE = 0.006, *p* < 0.001) per mL of plasma and urine, respectively [53].

A positive linear relationship between uTTV and pTTV was observed (Figure 1c): out of 301 observations, each 1 log copies/mL increase in uTTV corresponded to an increase in pTTV viral load of 0.56 log copies/mL (SE = 0.08, *p* < 0.0001). An increased TTV level was observed in men (plasma, 0.94 ± 0.48, *p* = 0.04; urine, 1.35 ± 0.48, *p* = 0.005). No association was found between plasma or urine TTV levels during follow-up and other patients’ baseline characteristics, types of administered induction, maintenance immunosuppressive therapy, or circulating biomarkers, except for an inverse association between pTTV and plasma creatinine. It is noteworthy that a correlation was observed between pTTV and uTTV and CMV viremia (*p* < 0.001 and 0.050, respectively).

### 2.3. TTV Viral Load and Infection Events

All subjects were monitored for infectious complications during follow-up.

To assess the association between TTV viral load and infection, we considered all episodes of infection, irrespective of the etiological agent or the severity of such episodes: both viral (including CMV infection or reactivation and VZV reactivation) and bacterial (with *Klebsiella pneumoniae* and *Escherichia coli* being the most commonly detected pathogens) infections, ranging from asymptomatic infections with positive cultures to severe infections such as urosepsis and enterocolitis.

We performed regression analyses to identify the association between events that occurred during follow-up (dependent variables) and TTV viral load (Table 7).

**Figure 2:**
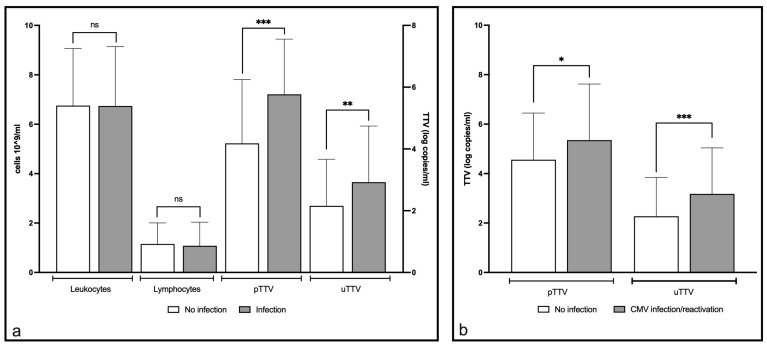
(**a**) differences between non-infection and infection events. (**b**) differences between non-infection and cases of CMV infection or reactivation (*t* test). ns: not significant, * *p* ≤ 0.05, ** *p* ≤ 0.01, *** *p* ≤ 0.001.

When all time points were considered, a higher pTTV was observed when clinical or laboratory signs of ongoing infection were present (mean: 5.735 log copies/mL; 95% CI: 5.392–6.079) compared to time points when no signs of infection could be detected (mean: 4.057 log copies/mL; 95% CI: 3.573–4.541; *p* value < 0.0001). Similarly, uTTV was significantly (*p* value = 0.0009) higher during ongoing infections than when no signs of infection were present (mean: 2.593 log copies/mL (95% CI: 2.143–3.042) versus 1.581 log copies/mL (95% CI: 1.169–1.993). The prevalence of infections increased across increasing quintiles of plasma and urine TTV levels (*p* < 0.0001 and *p* = 0.0005, respectively; Table 8). Interestingly, there were no differences in leukocyte or lymphocyte counts between the two groups (Figure 2a).

Six of 42 patients presented with a negative CMV serostatus at transplantation, 4 of them (66.6%) developed CMV infection; three of four patients presented symptomatic CMV disease (66.66%), with a registered viral load of up to 241,782 copies/mL. CMV reactivation occurred in 26 patients (61.9%) and was treated with valganciclovir, except in a few cases where spontaneous regression was observed. In patients with CMV reactivation or primary infection, an increase in TTV viral load was observed that preceded or coincided with peak CMV viremia. Interestingly, a statistically significant association was found between plasma CMV viral load and uTTV viral load (Figure 2b).

**Figure 3:**
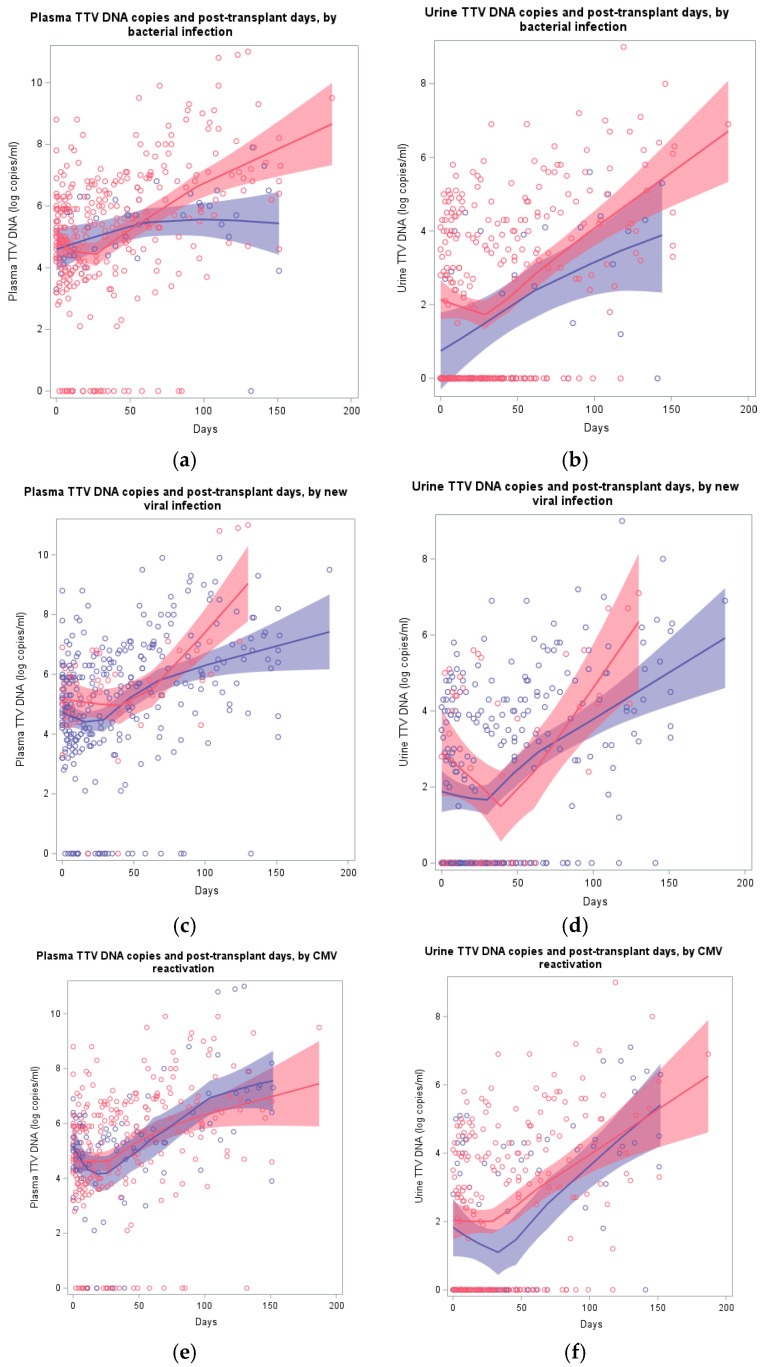
Distribution of plasma (**left**) or urine (**right**) TTV log copies by days from a bacterial (panel **a**,**b**) or viral (panel **c**,**d**) infection, or CMV reactivations (panel **e**,**f**) for subjects with (red) or without (blue) infections. Panel, plasma or urine, infection type and, for infection vs. no infections, total number of subjects, measurements and infection events: (**a**), plasma, bacterial, 35, 305, 75 vs. 7, 41, 0; (**b**), urine, bacterial, 35, 268, 66 vs. 7, 35, 0; (**c**), plasma, viral, 8, 54, 22 vs. 34, 292, 0; (**d**), urine, viral, 8, 47, 19 vs. 34, 256, 0; (**e**), plasma, CMV reactivations, 26, 255, 94 vs. 16, 92, 0; (**f**), urine, CMV reactivations, 26, 22, 86 vs. 16, 81, 0.

Figure 3 shows the distribution of pTTV or uTTV values according to days from transplantation, stratified for subjects who did or did not develop an infection during follow-up. The locally weighted regression shows a decreasing trend in the first 30 days, followed by an increase in plasma and urine TTV levels in the following months. The curves of both groups resulted to be similar and no statistically significant differences were observed comparing plasma or urine TTV values of subjects with vs. without an infection during follow-up. This result did not change in multivariate analyses using, beyond age and sex, immunosuppression and days from transplant as covariates, and did not change restricting the analysis to the first month or to the following months. Similarly, in the Cox regression analyses using infection at the following time point as an event, no statistically significant associations were found between plasma or urine TTV levels and the event.

**Figure 4:**
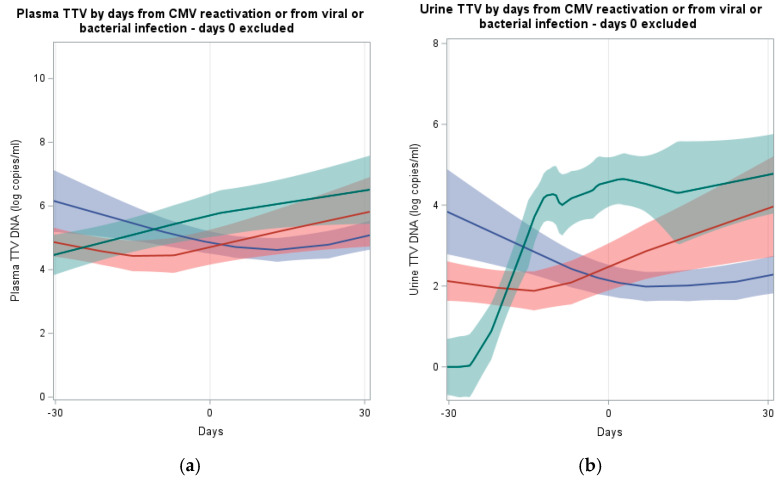
Plasma (panel **a**) or urine (panel **b**) TTV levels in subjects with a bacterial (blue) or viral (green) infection, or a CMV reactivation (red) during follow-up, according to distance from the day with the infection (day of positivity for infection has been fixed at 0 to align and center the curves); days 0 excluded. Total number of subjects, measurements, and infection events (panel **a**) plasma TTV levels, bacterial infections (35, 305, 75), viral infections (8, 54, 22), and CMV reactivations (26, 255, 94); (panel **b**) urine TTV levels, bacterial infections (35, 268, 66), viral infections (18, 47, 19), and CMV reactivations (26, 222, 86).

Figure 4 showed the distribution of plasma or urine TTV according to distance from the infection day, in the subgroup of subjects who developed an infection, to better focus on the time trend of TTV levels within subjects who developed a specific infection. The day of infection positivity was fixed at day 0 and the TTV curves of each subject, for the three infection types, were thus aligned and centered at their day 0. The locally weighted regression analyses showed that subjects with a new viral infection, in the days before the infection, reported an increase in both plasma and urine TTV viral load. An increase has been observed also in the case of CMV reactivation, starting 7 days before the event, while in the case of bacterial infection, an increase has been observed starting 15 days after the infection event. Although the increasing trends in the days before the infection suggest a potential use of TTV levels as predictors of new viral infection events or a CMV reactivation, the number of subjects was low to conduct an analysis of predictive abilities.

## 3. Discussion

TTV has been proposed as a marker to assess the immune status of SOT recipients, and it is believed that it may be useful to modulate the immunosuppressive load and prevent complications due to insufficient or excessive immunosuppression, such as rejection and opportunistic infections. It is known that patients with a compromised immune system, such as KT recipients, have an increased TTV plasma load. Several studies have found a positive association between TTV load and risk of infection and a negative association between TTV load and risk of rejection [36]. Most of these studies focus on the role of plasma TTV load, but not on the role of urinary TTV load.

In recruited patients, in the immediate pre-transplant period, the mean pTTV viral load was 5.30 log copies per mL plasma, while only three patients provided a pretransplant urine sample, and there was no evidence of urinary excretion of TTV in any subjects with residual diuresis. A decrease in pTTV was observed immediately after KT. A decrease in viremia in the immediate post-transplant period is also described in previously published studies and appears to be justified by the site of viral replication, consisting of certain subpopulations of peripheral blood T lymphocytes, and by the lymphopenicising effect of induction therapy [52,53]. In contrast to other studies published in the literature, which described an earlier increase in viral titers around day 20 post-KT, in this study, an increase in pTTV was observed from day 30 post-KT.

In the first hypothesis, these differences can be attributed to the cohort of patients considered; in fact, most studies describing TTV kinetics published to date have recruited either a pediatric population or lung or combined kidney–pancreas transplant recipients, in whom the kinetics of TTV are different due to differences in the immune system and immunosuppressive regimen. In the second analysis, there are differences related to the immunosuppressive therapy administered, as more than 10% of the recruited subjects were receiving their second or third KT, and several patients had a high number of panel reactive antibodies (PRA), necessitating the use of dual induction therapy with basiliximab and thymoglobulin, which is known to be more lymphopenic than basiliximab alone.

There is a paucity of data in the literature regarding the role of urinary TTV load. Our data demonstrated that the viral load of uTTV follows similar kinetics to that of pTTV, with an initial decrease and an increase at the end of the first month.

Similar trends in pTTV and uTTV kinetics are justified by the existence of a positive linear relationship between pTTV and uTTV: each increase of 1 log copies/mL in uTTV corresponds to an increase of 0.55 log copies/mL in pTTV viral load (*p*-value < 0.0001). No study published to date has described the kinetics of the urinary viral load of TTV in KT recipients, so comparisons could not be made.

The increase in viral load after transplantation seems to reflect the increasing degree of immunosuppression after transplantation until stabilization occurs towards the end of the third month. This appears to be in line with the current consensus that the first three months after transplantation are the most critical period, when there are greater fluctuations in the concentration of immunosuppressive drugs, resulting in a greater risk of complications.

Because of its correlation with immune system function, Torque Teno Virus has been proposed as a prospective marker to predict infection and rejection in SOT recipients. All subjects enrolled in this study were followed to assess any episodes of rejection and infectious complications that occurred during follow-up. It was not possible to assess the endpoint related to rejection, as no biopsy-confirmed episodes of rejection occurred in any of the subjects enrolled in the study during the entire follow-up period.

For each time point, weekly, or even more frequently in the immediate post-transplant period, we evaluated the presence of clinical and laboratory signs suggestive of infection, regardless of clinical manifestation or etiological agent, and observed that pTTV load was 40 times higher (*p* value < 0.0001) when infection was ongoing compared to time points when the patient did not manifest signs of infection. Similarly, there was a statistically significant association (*p* < 0.0001) between urinary TTV load and the presence of infection, with a 10-fold higher urinary TTV load in ongoing infections. Indeed, there are known associations between TTV levels and infectious events; Doberer and coworkers [54] report that each increase of 1 log copies/mL corresponds to an 11 percent increase in infectious risk (*p* < 0.001), while Fernández-Ruiz and coworkers highlight that there is already increased risk of developing opportunistic infections at a viral titer of 3.15 log copies/mL [55]. A meta-analysis by van Rijn and coworkers [38] reports how different studies identify different cut-offs at different time points after transplantation, making it necessary to identify critical values at predetermined time points or to create a model that can be applied regardless of the time elapsed since transplantation to assess infectious risk. Since we observed no differences considering distance from transplantation, while we found a changing trend in days before viral infections, we suggest considering changes over time in the same subjects, irrespective of time distance from transplantation. Our study suggests to perform combined measurements of plasma and urine TTV levels, to potentially predict new viral infections or CMV reactivations, one of the major infectious agents causing morbidity and mortality in transplant recipients [56]. However, large studies measuring both plasma and urine TTV levels, along with FK, at frequent time points and for a long follow-up should be conducted to study predictive abilities of TTV levels.

## 4. Materials and Methods

A total of 42 consecutive patients who underwent kidney transplantation at the Transplant Centre of the Ospedale di Circolo and Fondazione Macchi in Varese, Italy, between September 2022 and March 2023 were enrolled in this prospective study. All enrolled KT recipients fulfilled the following inclusion criteria: (a) KT was performed in our center, (b) regular follow-up was performed in our outpatient clinic for at least the first 3 months after KT, (c) only patients initially treated with maintenance tacrolimus (FK), mycophenolate (MMF), and steroids (CS) were included. Exclusion criteria were: (a) transplantation and/or initial treatment in another center and (b) maintenance treatment other than allogeneic hematopoietic cell transplantation. Ethical approval for this study was obtained from the Ethical Committee of University of Insubria—approval number 74724.

Post-transplant care was provided according to the standards of our center and KDIGO 2009 guidelines. All patients received peri-HCT induction therapy selected according to donor and recipient risk factors: Basiliximab and steroids were administered to all patients, while only high-risk patients were additionally treated with rATG. MMF was started on the first post-KT day, while FK was usually started on days 4–5. During follow-up, plasma tacrolimus trough levels were monitored, and tacrolimus doses were adjusted if out of range.

In accordance with our center’s standard of care, no protocol graft biopsies were performed, and no alloantibodies were measured. No patient showed signs of acute rejection during follow-up. Allograft rejection, defined as graft dysfunction and failure due to the recipient’s immune response to the genetically different graft, is diagnosed by an initial clinical evaluation and laboratory tests designed to assess renal function, creatinine, and urea in the first place. If rejection is suspected, donor-specific anti-HLA antibodies (DSA) are evaluated, and ultrasound and renal biopsy (the gold standard for certainty of diagnosis) are performed.

All patients were closely monitored for post-transplant infections.

No patient was treated with CMV prophylaxis according to our center’s standards, as CMV viral load monitoring is currently in place to inform preemptive treatment with antivirals. The CMV viral load above which antiviral therapy is given is 3000 copies/mL, and in case of rapid rise, therapy is started at lower values, 2000 copies/mL.

All patients received trimethoprim/sulfamethoxazole chemoprophylaxis for the first six months after KT.

Plasma and urine samples were collected before KT and at selected time points, weekly, or even more frequently in the immediate posttransplant period, during the first 100 days after KT. TTV levels were compared with clinical data from the patients’ medical records, microbiological analysis, and blood tests (including trough FK levels, serum creatinine, and blood count). Plasma and urine samples were collected and stored at −80 °C until processed.

Automated DNA extraction was performed using BioerNPA-32P (Bioer Technology, Hangzhou, China) and MagaBio plus Virus DNA/RNA Purification Kit II (Bioer Technology). Extracted DNA was quantified fluorometrically using Invitrogen (Waltham, MA, USA)—Qubit 4 and stored at −80 °C. The extracted DNA was amplified by real-time PCR using TaKaRa Premix Ex Taq ™ (Takara Bio Group, San Jose, CA, USA) and Applied Biosystems Taqman universal PCR master mix (Thermo Fisher Scientific, Waltham, MA, USA). Primers and probe were designed from a portion of the untranslated region (UTR), which was found to be highly conserved among all TTV sequences available in the GenBank at the time of writing. The oligonucleotide sequences are as follows: AMTS (forward primer 5′-GTGCCGIAGGTGAGTTTA-3′, position 177-194), AMTAS (reverse primer 5′-AGCCCGGCCAGTCC-3′, position 226-239), and AMTPTU (TaqMan probe 5′-TCAAGGGGCAATTCGGGCT-3′, position 205-223). The probe was labeled by 6-carboxy-fluorescein (FAM) and 6-carboxy-tetramethyl-rhodamine (TAMRA) at its 5′ and 3′ ends, respectively. Each run contained several negative controls (no template) as well as the reference template (positive control) at 10^1^ to 10^6^ copies/10 µL. Procedures for quantification of copy number and evaluation of intra- and inter-assay precision and reproducibility of the assay have been previously reported. The lower limit of sensitivity of the assay was 1.0 × 10^1^ copies per mL of plasma or urine.

The post-transplantation kinetics of TTV were analyzed using the MEANS procedure of the SAS software, quantifying for each timepoint the mean plasma concentration of creatinine and FK and the plasma and urinary viral loads of TTV.

Descriptive statistics were performed to report the general characteristics of the population. To report an estimate of trends for the TTV DNA copies during time, a locally weighted regression has been used, using a scatterplot smoothing method that automatically determines the optimal smoothing parameter (PROC SGPLOT with LOESS statement in SAS). The relationship between clinical factors and plasma or urinary viral load of TTV was analyzed by multiple regression analysis, using baseline levels of pTTV or uTTV as the dependent variable and recipient’s age at transplantation, recipient’s sex, delayed graft function, years of dialysis, induction therapy, and maintenance therapy with FK, as the independent variables. Then, the same analyses were performed using baseline and follow-up pTTV and uTTV levels as dependent variables and plasma creatinine, lymphocytes, leukocytes, tacrolimus trough levels and days since transplantation as independent variables. For these last analyses, the repeated subjects option was used in PROC GENMOD of the SAS software to take into account the repeated measurements performed in the same subjects. The analyses were then replicated, adding days since transplantation and variables found statistically significant associated with TTV levels in previous analyses, as covariates. Finally, the same analyses were performed using DGF or infection during follow-up as dependent and TTV as independent variables. We further performed a Cox regression analysis considering all covariates as time-dependent, and first incident infection occurred during follow-up, at the next measurement day, as event (PROC PHREG with count method for time-dependent covariates in SAS software). This method deals with the undue increase in statistical power of an analysis of each repeated measurement of TTV values in the same subjects. The variable TTV DNA copies have been log-transformed, using the formula ln(number of copies plus 1), to deal with the problem of log(0) in case of negativity for TTV. The statistical analyses were performed using the SAS software 9.4 (v9.4, SAS Institute Inc., Cary, NC, USA) or by means of the GraphPad software Prism 9.

## 5. Conclusions

The number of studies delineating the role of TTV as a marker of immunosuppression is increasing. There is evidence that TTV is useful not only in estimating the level of immunosuppression, but also in predicting the occurrence of potential complications. This is because TTV seems to reflect overall immune function.

While plasma TTV levels at T0 were associated with dialysis years, no association was found between plasma and urine TTV levels during follow-up or in other patients’ baseline characteristics, type of administered induction, or maintenance immunosuppressive therapy, or circulating biomarkers, except for an inverse association between pTTV and plasma creatinine, lymphocytic count, and leukocytes. It is noteworthy that an elevated TTV level was observed in males.

This study demonstrated a positive linear relationship between pTTV and uTTV, and that quantification of the virus not only in plasma but also in urine is a relevant marker of immune status. Both samples can be used as a guide for modulating immunosuppressive therapy as well as to predict the onset of infectious complications. Of particular interest is the finding of a strong correlation between urinary TTV levels and CMV infection or reactivation. This may pave the way for the selective use of urinary TTV to monitor CMV reactivation. This is of particular importance as in these patients, leukocyte or lymphocyte counts are not reliable.

Given that no differences were observed in relation to distance from transplantation, while a changing trend was identified in days before viral infections, it is recommended that changes over time in the same subjects, irrespective of time distance from transplantation, be considered.

Further studies in larger populations are needed to assess the timing of pre-infection TTV elevation and to evaluate predictive metrics, as well as the identification of predetermined time points, cut-offs, and the development of validated and easily applicable models.

## Figures and Tables

**Table 1:** Population characteristics.

Variable	N	Mean	SD
Age (years)	42	54.7	12.1
Follow-up days	42	104.9	38.5
Dialysis years	42	4.39	3.52

**Table 2:** Population characteristics. CMV: cytomegalovirus, rATG: rabbit antithymocyte globulin.

	N	%
Men	24/42	57.1%
Kidney disease:		
Glomerular disease	13/42	28.6%
Cystic disease (ADPKD)	8/42	19.0%
Diabetic nephropathy	4/42	9.5%
Hypertensive nephrosclerosis	4/42	9.5%
Nephrectomy	5/42	11.9%
Chronic pyelonephritis in VUR	3/42	7.1%
Others	5/42	11.9%
Positive CMV serostatus at baseline	37/42	88%
Living donor transplant	8/42	19%
Previous transplants	7/42	16.6%
Double induction therapy (Basiliximab + rATG)	13/42	31.0%
Delayed Graft Function (DGF)	14/42	33.3%

**Table 3:** Population immunological characteristics. PRA: panel-reactive antibody; MM: mismatch number.

Induction Therapy	N	PRA% (Mean ± SD)	MM (Mean ± SD)	pTTV (Mean ± SD)	uTTV (Mean ± SD)
Basiliximab + rATG	13	15 ± 0.13	6.3 ± 2.69	5.58 ± 2.09	3.16 ± 1.63
Basiliximab	29	8 ± 0.08	5.6 ± 2.16	5.6 ± 1.34	3.55 ± 1.8

**Table 4:** Association between baseline pTTV DNA copies (dependent variable) and baseline characteristics (generalized regressions for repeated measures, age and sex as covariates). SE: standard error.

Parameter	N	Estimate	SE	*p*-Value
Age	26	0.02	0.02	0.31
Sex (men)	26	−0.11	0.50	0.83
Dialysis years	26	0.21	0.07	0.005
Creatinine (mg/dL)	26	−0.05	0.10	0.62

**Table 5:** Medium plasmatic creatinine, tacrolimus (FK), pTTV, and uTTV at selected time points.

Day	Number of Measurements at the Selected Time	Creatinine (mg/dL)	FK (ng/mL)	pTTV (log copies/mL)	uTTV (log copies/mL)
0	41	7.39 (±2.64)	-	5.26 (±1.31)	1.30 (±1.83)
15 (11–20)	46	4.38 (±3.04)	11.16 (±5.06)	4.54 (±1.77)	2.17 (±2.04)
25 (21–30)	33	3.009 (±2.48)	9.59 (±3.04)	4.33 (±2.13)	1.49 (±1.87)
35 (31–40)	25	2.17 (±1.44)	9.37 (±3.13)	4.40 (±2.49)	1.70 (±2.31)
45 (41–50)	23	1.80 (±0.69)	9.61 (±2.99)	4.93 (±1.99)	1.85 (±1.96)
55 (51–60)	15	1.58 (±0.41)	8.81 (±3.22)	5.76 (±2.11)	2.78 (±2.37)
65 (61–70)	23	1.61 (±0.49)	9.77 (±3.16)	5.90 (±1.97)	3.09 (±2.11)
75 (71–80)	11	1.78 (±0.99)	8.78 (±1.65)	7.03 (±1.57)	4.45 (±2.08)
85 (81–90)	12	1.73 (±0.97)	10.58 (±5.67)	5.67 (±3.13)	3.02 (±2.47)
95 (91–100)	11	1.62 (±0.36)	8.65 (±1.37)	6.14 (±1.24)	4.00 (±1.98)
>100	43	1.54 (±0.53)	9.02 (±2.17)	7.00 (±2.08)	4.64 (±2.04)

**Table 6:** Associations between plasma or urine TTV DNA copies, measured at baseline and follow-up, and clinical and laboratoristic parameters. Generalized regressions for repeated measures, age, and sex as covariates.

	Plasma TTV			Urine TTV		
Parameter	N	Est ± SE	*p*-Value	N	Est ± SE	*p*-Value
Age	346	0.02 ± 0.02	0.16	303	0.02 ± 0.01	0.06
Sex (men)	346	0.94 ± 0.48	0.04	303	1.35 ± 0.48	0.005
FK trough levels (ng/mL)	305	0.04 ± 0.05	0.42	281	−0.02 ± 0.06	0.75
Dialysis years	346	−0.05 ± 0.06	0.46	303	−0.07 ± 0.07	0.33
Leukocytic count (10^9^/mL)	261	−0.07 ± 0.08	0.34	239	−0.06 ± 0.08	0.45
Lymphocitic count (10^9^/mL)	258	0.23 ± 0.32	0.47	236	0.40 ± 0.26	0.13
Creatinine (mg/dL)	343	−0.15 ± 0.05	0.007	301	−0.18 ± 0.07	0.01
CMV viral load (log)	275	0.15 ± 0.04	<0.0001	249	0.12 ± 0.06	0.053
Double induction therapy	346	0.62 ± 0.51	0.22	303	0.20 ± 0.52	0.70
Days elapsed since KT	346	0.02 ± 0.004	<0.0001	303	0.02 ± 0.004	<0.0001
uTTV	301	0.56 ± 0.08	<0.0001	-		
Days since KT *	346	0.02 ± 0.004	<0.0001	303	0.02 ± 0.004	<0.0001
uTTV *	301	0.56 ± 0.08	<0.0001	-		
CMV viral load (log) *	275	0.15 ± 0.03	<0.0001	227	0.12 ± 0.06	0.050

* adjusted for number of years the patient underwent dialysis and type of induction therapy, single or double induction with rabbit anti-thymocyte globulin (rATG).

**Table 7:** Associations between plasma or urine TTV DNA copies, measured at baseline and follow-up, and events during follow-up. Generalized regressions, age, and sex as covariates.

	Plasma TTV (Log Copies)				Urine TTV (Log Copies)			
	N	Days	Est ± SE	*p* Value	N	Days	Est ± SE	*p* Value
CMV	275	115	0.06 ± 0.01	<0.0001	249	105	0.06 ± 0.02	0.003
Infection (±1 day) *	346	160	0.04 ± 0.01	0.002	303	146	0.05 ± 0.01	0.0003
Bacterial infection	346	40	0.00 ± 0.01	0.84	303	38	0.02 ± 0.01	0.007
Viral infection	346	19	0.01 ± 0.01	0.13	303	17	0.01 ± 0.01	0.88
CMV reactivation	339	94	0.04 ± 0.01	0.012	296	86	0.04 ± 0.02	0.015

* positive day: positivity on that day or on the previous and following day when unmeasured; days without measurements have been considered as negative days.

**Table 8:** Prevalence of infection days across quintiles of plasma and urine TTV DNA levels.

	pTTV			uTTV		
	No Inf	Infection	Total	No Inf	Infection	Total
Lowest quintile	51	20	71	82	42	124
	71.8%	28.2%	20.5	66.1%	33.9	40.9%
2nd quintile	39	27	66	15	11	26
	59.1%	40.9%	19.1	57.7%	42.3	8.6%
3rd quintile	42	25	67	32	20	52
	62.7%	37.3%	19.4	61.5%	38.4	17.2%
4th quintile	45	31	76	21	30	51
	59.2%	40.8%	22.0	41.2%	58.8	16.8%
Highest quintile	25	41	66	21	29	50
	37.9%	62.1%	19.8%	42.0%	58.0	16.5%
Total	202	144	346	171	132	303
	58.4%	41.6%	100	61.9%	38.1	100%

## Data Availability

The data presented in this study are available on request from the corresponding author.

## Supplementary Materials

The following supporting information can be downloaded at: https://www.mdpi.com/article/10.3390/ijms25147744/s1.
